# Martyrs of the Age

**Published:** 1859-09

**Authors:** 


*Martyrs of the Age.*

If any "used up" gentleman who has fruitlessly exhausted himself day after day in meditation, or in aught else, at the bow window of his club, in expectancy of a "new sensation," would apply to us, we think we could assist him as regards the object of which he is in search. We warn him, however, that we could not allow him to be particular, and that so long as we treated him to something both novel and of decided *gout*, he would have to be content with his dish. But we could promise him this, that our "new sensation" should be one he had never felt before, and that. if he continued to indulge in it with but a small amount of constancy, he should, if made at all of penetrable stuff, for the future contemplate society from—to him—a very profitable, though peculiar point of view. That he must not be squeamish, we caution him: for however correct Archdeacon Hale may have been in reminding us that death is precisely as natural as life, we certainly do not so much seek its contact. And with death and disease we should have to work; in fact, we should hurry our friend from all those delicate and refined usages of the world which surround him, but which he has, nevertheless, found so "stale, flat, and unprofitable," and stop only when we had reached those grim abodes of misery and labor whose inhabitants have been dowered with a short life, which is nothing after all but a long dying. Our duty would be to show him that gaunt demon of suffering who presides over the many applications of human industry to the conveniences and the luxury of our time.

But lately a despot died; the jubilates for the event have scarcely ceased their sound at Naples ere the Court of a free and Protestant country proclaims a solemn miserere for it by what is termed "going into mourning." Admirable sympathy! but that is not our present point. We have to take our friend from St. James' to the millinery factories of the chief city of the world. Why? To show that when all the Court of England goes into mourning, four dozen girls are rendered blind for life, so trying to the eyes is the immense labor on black lace which such an event entails! Reader, does this startle you? We would give our friend the opportunity for further disclosures in connexion with lace. For instance, some of the choicest kinds require a thread so fine that it must be spun and worked in damp cellars—a process which costs annually hundreds of female lives. Not many months since, a young woman, employed in lace cleaning, died in Paris under circumstances which necessitated a judicial inquiry. The post mortem examination of the poor creature's body revealed the presence of large quantities of oxydized lead in the system. She had worked at cleaning lace by the "Belgian process," a process in which the lace is whitened by repeatedly dusting it with "white lead." From the time it was spun in the damp cellar to its revivification from the yellowness of years, would our friend find the material of his Court ruffles too often but a beautiful luxury entailing suffering and death. True is it that, but few are permitted to regale themselves in this costly garniture of fine and courtly lace.

But who does not rejoice in "lucifer matches," "congreves," or other spontaneous inflammables composed of phosphorous? Let us go to Whitechapel, if our friend can possibly for once go east of "Wussell Square." That classic region attained, let us enter a factory. Strange place it is—in some parts how terribly draughty! well it is so, or the fumes from the drying matches would be concentrated poison. But what is that miserable, mumbling creature about who is alternately stooping towards a pot and placing his hand against his jaw? His occupation all day is to sit over a pot of melting glue and other ingredients, and to cut sticks of phosphorus into the size of a pea, and to throw them one by one into the glue. And from what does he suffer? Why from incurable disease, or total destruction of his lower jaw bone. For the sake of employment and at good wages, he begins exposing himself to the fumes of phosphorus acid with a rotten tooth or two in his head. In no long space of time, he is seized by toothache and annoyed by gumboils. Abscesses follow, and his teeth drop out. But he works on, until he has nothing left but "a rotting and diseased periosteum, and a jaw bone as dead and as dry as one might see in a churchyard, for it is not at all like caries or necrosis." Can we wonder at what the "Annales d'Hygiene" tells us, that in France the laborers at this dangerous employment are dissipated in their habits, irregular in their attendance, and recruited from the lowest class? True it is, the "dippers" in some factories wear sponges before their mouths, and the work people are required to wash their hands night and morning in a solution of soda. Some careful workmen without bad teeth luckily escape altogether, if the ventilation be very good; others are infinitely less fortunate. We have heard of a young man who, laboring under the effeets of the fumes of phosphorus, presented himself for examination. Although he had not been engaged in the manufacture of lucifers for eighteen months, ye yet smelt so strongly of phosphorus that he impregnated the atmosphere of the room. He had never taken a bath, and, from his extreme poverty, had probably worn the same clothes for eighteen months.

Let us visit one of the workrooms which are kept up during the London season to meet the "instantaneous demands" upon the fashionable tailor. There it is, sixteen or eighteen yards long, and seven or eight yards wide. Eighty men are packed together, working knee to knee. They must have candles even in summer, for they work late. What with the heat of the men, the heat of the irons, and the heat of these candles, the mephitic air is twenty to thirty degrees higher than it is outside—and it is summer time too! The fresh tailors from the country faint away, and they complain of the heat and smell as intolerable. The men are sitting as loosely dressed as possible, the perspiration streaming from them. On what they call the cold nights the room is so hot that, large thick tallow candles (quarter of a pound candles) have melted and fallen over from the heat. The young hands are unable to work full time; the old hands lose appetite: thirst takes the place of hunger, and gin of food!

But not alone do lace makers and milliners, workers with phosphorus, and tailors, pursue their callings, the source of their own sustenance and of the luxuries and conveniences of the world, under circumstances which hurry them to an early grave. Scores of arts are thus burdened with fearful penalties to those who practice them; and though man pushes on with new inventions, he is reckless of the results of the old, only caring, indeed, to show what further brilliant and startling novelties can be produced to further the luxurious disposition of the age. What is the history of a London workshop? Dr. Guy shall tell us:

"A man begins by employing a few hands in a house, often ill adapted for an ordinary dwelling house; and as his business increases he contrives to add one low apartment to another, by knocking down partition walls and making such alterations as suit his immediate purpose. He contrives by this means to accommodate an increasing number of men, and the only practicable limit to that number is the want of more standing or sitting room, as the case may be. He warms these rooms by a stove, by steam, or by hot air and lights them with gas; the consequence is that the workmen are exposed at the same time to a high temperature and a foul and stagnant atmosphere. This combination is carried to its highest degree in the tailors' workshop; and I have been told, more than once, by the journeymen tailors themselves, that they have been obliged to strip to the very skin, that they might be able to bear the intense heat to which they are exposed. In buildings intended for workshops, more space are given to the men; but they are usually constructed on very bad principles. The whole building often forms one space, divided by floors perforated by a common staircase; if a steam engine is employed, it is generally to be found in a lower apartment of the building, so that the heat rises from this into the upper rooms, and mingling with the foul air of the intermediate floors, ascends to the highest flat, where the heat and foul air collect in great abundance."

From such places as these come all the more complex and important offerings that applied science can make to the refined requisitions of the day.

Let us change the scene, and go where misery and starvation create a science of their own. We stop at the "Ruins" near Turnmill Street, Old Field Lane. In a wretched alley there is a garret where lives a widow and four children. The room has a curious aspect, for its corners are filled with scraps and fragments of paper, rags, and cloth of every shape and color, which the children are sorting out into separate heaps, while the widow superintends them, and works herself. The whole family gain their living, if such slow death deserves that term, by rising early in the morning, (when the widow and her children go forth,) and, each taking a certain district, wait till the city warehouses are swept out, when they carefully watch and gather up the rubbish of paper and rags which are cast into the street. Until noon all are thus occupied, when each returns with a little bundle to the garret where they dwell, and they pass the remainder of their time in sorting out and drying the proceeds of their labor. On a fine day, by such incessant labor, this widow and her children can earn *ninepence!* In wet or windy weather the most strenuous exertions scarcely produce sixpence, and but for those humble societies which distribute coals and bread amongst such hapless beings, they would literally starve outright.

Now, we have afforded a hint to him who is *ennuye* and desperate for a "new sensation" how he may rejoice in one if he will take the trouble to go the right way for it. Only let him follow for a day or two the working bees of our social hive, and if all feeling has not been swamped in the sluggish pool of fashionable nonchalance, we are much mistaken if he will not experience some strange "visitings of conscience" before his walk is done. Should he be desirous of novelty of action, as well as of sensation, of something to do as well as of something to feel, why then let him devote part of his idle time to the good of his fellow creatures by thus following the progress of the arts and of human ingenuity, only that they may endeavor to lessen the sufferings which they bring with them in their train. They are many in number, often dreadful in character, and present a wide field of action for the philanthropist who would read aright the history of the REAL MARTYRS OF THE AGE!—*London Lancet.*

